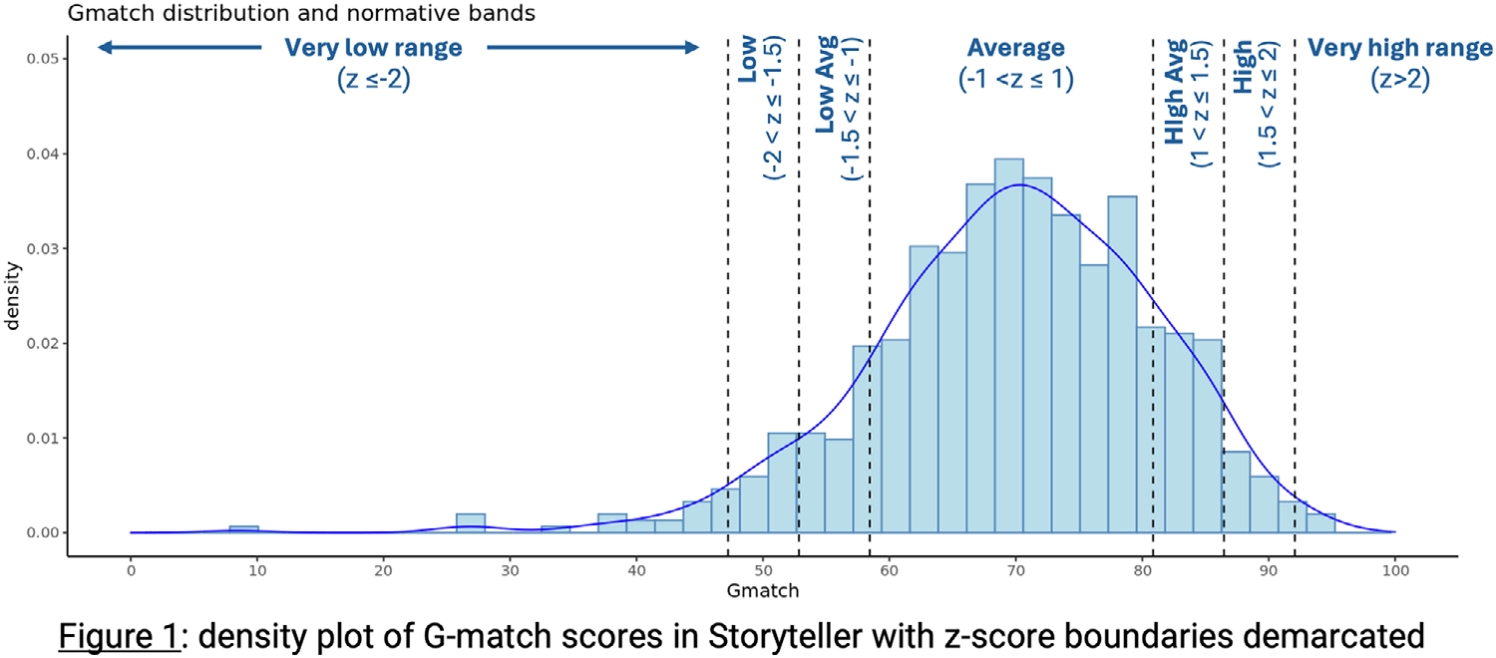# Storyteller: Normative data for the brief, remote, unsupervised automated speech‐based AD/MCI clinical trial screening measure

**DOI:** 10.1002/alz.093153

**Published:** 2025-01-03

**Authors:** Caroline Skirrow, Udeepa Meepegama, Michael T. Ropacki, Jack Weston, Emil Fristed

**Affiliations:** ^1^ Novoic, London United Kingdom; ^2^ Strategic Global Research & Development, Temecula, CA USA

## Abstract

**Background:**

Storyteller, a brief self‐administered test that uses speech analysis to measure cognitive functioning, has demonstrated ability to predict Mild Cognitive Impairment (MCI) and mild Alzheimer’s disease (AD). The test is being implemented globally in Sponsor clinical trials, ADNI, and Sites for pre‐screening and will be used across heterogeneous populations. Normative data for Storyteller exists and is important for contextualising test performance, but has not been previously published.

**Method:**

US residents aged 50+ were recruited into the AMYWEB study (NCT05298501). Participants completed a remote assessment via web application on their own personal devices, including screening and demographic questionnaires, the Patient Health Questionnaire‐8 (PHQ8), and Storyteller. Participants’ spoken responses were recorded, automatically transcribed and analysed to obtain a measure of proportional recall, “G‐match”, that leveraged two immediate and one delayed story recall conditions. Linear regression examined the influence of age, education, and sex on G‐match. Z‐scores were calculated to determine normative task performance ranges.

**Result:**

679 participants (381 females, 298 males; age range 50‐84, mean 59.52) were recruited after excluding individuals with incomplete submissions, history of mild cognitive impairment (MCI), dementia or stroke and PHQ‐8≥10. The sample was demographically diverse, with residents across 49 US states and representation from minoritized groups (n = 115 overall: Black or African American n = 66; Hispanic or Latino/a n = 28; Asian n = 22; Native American or Alaska Native n = 18; Pacific Islander n = 4)). Assessments were completed across a range of common devices and browsers. Z‐scores identified score bands across different performance ranges (Fig 1). Regression analyses showed no significant age‐related change (β = ‐0.07, p = 0.21), but revealed higher scores for more educated participants (β = 0.80, p<0.001) and lower scores in males (β = ‐3.00, p<0.001).

**Conclusion:**

Storyteller’s remote, unsupervised completion across a diverse U.S. sample generated valuable normative data, allowing contextualization of how impaired recruited samples are within AD/MCI clinical trials and in the broader population. Although G‐match showed no meaningful decline with age, the current findings replicate research showing a protective effect of education on cognitive function, and modestly better performance by women for story recall tasks.